# Pantoprazole Does not Affect Serum Trough Levels of Tacrolimus and Everolimus in Liver Transplant Recipients

**DOI:** 10.3389/fmed.2018.00320

**Published:** 2018-11-19

**Authors:** Sebastian C. B. Bremer, Lars Reinhardt, Michael Sobotta, Marie C. Hasselluhn, Thomas Lorf, Volker Ellenrieder, Harald Schwörer

**Affiliations:** ^1^Clinic for Gastroenterology and Gastrointestinal Oncology, University Medical Center Goettingen, Georg-August-University, Goettingen, Germany; ^2^Liver Center Goettingen, University Medical Center Goettingen, Georg-August-University, Goettingen, Germany; ^3^Clinic for Anesthesiology, University Medical Center Goettingen, Georg-August-University, Goettingen, Germany; ^4^Clinic for General, Visceral and Pediatric Surgery, University Medical Center Goettingen, Georg-August-University, Goettingen, Germany

**Keywords:** immunosuppressive drugs, pantoprazole, serum trough levels, liver transplant recipients, tacrolimus, everolimus

## Abstract

**Background:** Liver transplant recipients are frequently treated with proton pump inhibitors. Drug interactions have been described especially with respect to omeprazole. Due to the lower binding capacity of pantoprazole to CYP2C19 this drug became preferred and became the most used proton pump inhibitor in Germany. The data on the influence of pantoprazole on immunosuppressive drugs in liver transplant recipients a very scarce.

**Methods:** The authors performed a single center analysis in liver transplant recipients on the effect of pantoprazole on the serum trough levels of different immunosuppressants. The trough levels were compared over a period of 1 year before and after start or stop of a continuous oral co-administration of 40 mg pantoprazole once daily.

**Results:** The serum trough levels of tacrolimus (*n* = 30), everolimus (*n* = 7), or sirolimus (*n* = 3) remain constant during an observation period of at least 1 year before and after co-administration of pantoprazole. None of the included patients needed a change of dosage of the observed immunosuppressants during the observation period.

**Conclusions:** The oral co-administration of pantoprazole is safe in immunosuppressed liver transplant recipients according to the serum trough levels of tacrolimus, everolimus, and sirolimus. This analysis provides first data on the influence of pantoprazole on immunosuppressive drugs in liver transplant recipients.

## Introduction

Proton pump inhibitors (PPIs) are frequently used in non-transplanted liver cirrhotic patients (1). The reasons for use of PPIs are broad: primary prevention of gastrointestinal ulcer or bleeding, gastrointestinal inflammation (e.g., gastritis), gastroesophageal reflux disease and secondary prevention e.g., after bleeding of esophageal varices (2–5). Data on the use of PPIs in liver transplant recipients are very rare.

Drug interactions of PPIs have been described during the last years, especially about omeprazole (6, 7). Omeprazole is strongly metabolized by cytochrome P450 enzymes (CYP), especially by CYP2C19 and less by CYP3A4 (8–11). The metabolizing process leads to a competitive inhibition of these enzymes which might affect the metabolism of other drugs like the immunosuppressive drugs tacrolimus, everolimus and sirolimus which are also metabolized by CYP3A4 (7). The combination of omeprazole with the immunosuppressive agents cyclosporin A as well as tacrolimus in liver transplant recipients might lead to side effects due to increased serum levels e.g., nephrotoxicity, neurotoxicity and post-transplant diabetes mellitus (12–18).

The PPI Pantoprazole is also mainly metabolized by CYP2C19 and CYP3A4 (6, 7, 19). It is the most prescribed PPI in Germany (20). Pantoprazole did not influence the serum levels of cyclosporin A in kidney transplant recipients (21). Rančić et al. described lower serum trough levels of tacrolimus in kidney transplant recipients (22). Data on the influence of pantoprazole on the serum trough levels of tacrolimus in liver transplant recipients are scarce. The only data in liver transplant recipients are presented by Lorf et al. who did not observe any drug interactions of pantoprazole and tacrolimus in a very small group of only two liver transplant recipients (23). There are no data on the influence of pantoprazole on the serum levels of everolimus or sirolimus with focus on liver transplant recipients.

Due to the scarce data on the influence of the most used PPI in Germany pantoprazole we performed a single center analysis on the effect of pantoprazole on the serum through levels of tacrolimus as well as on everolimus or sirolimus in a cohort of liver transplant recipients.

## Materials and methods

### Patient selection

We analyzed the patient records of all adult liver transplant recipients (178 patients) that have been regularly monitored on an outpatient basis at the University Medical Center Goettingen between February 2009 and January 2015.

We determined time points when a continuous oral pantoprazole therapy with 40 mg once daily was initiated or stopped. All included liver transplanted patients had regular follow-up appointments at our center at least every 6 months. Blood analyzes including measurements of the serum trough levels of immunosuppressive agents were performed on all appointments. The included patients were observed in outpatient basis and advised to the correct and timely intake of their drugs at all contacts.

The following patients were excluded from our retrospective analysis: (1) Patients with discontinuous use of pantoprazole or the application of pantoprazole in lower or higher dosages than 40 mg once daily. (2) Patients with a change of dosage of potentially interacting co-medications (e.g., amlodipine) based on prescribing information and interaction database (Medscape, https://reference.medscape.com/drug-interactionchecker). (3) Patients with an acute or chronic cholestasis, because immunosuppressive drugs like tacrolimus or everolimus are eliminated via the biliary system (24, 25).

Finally, we could identify 40/178 (22.5%) liver transplanted patients who fulfilled the above-mentioned criteria. We compared the serum trough levels of the immunosuppressive drugs in these patients with respect to the absence or presence of oral co-administration of 40 mg pantoprazole once daily. Therefore, we analyzed the first measured serum trough levels before and after change of PPI co-administration and further analyzed all routine measurements over a period of 52 weeks or more before and after change of co-administration.

### Analysis of serum levels of immunosuppressive drugs

The serum for the measurements of immunosuppressive drug levels was taken in the morning between 8 and 9 a.m. before intake of the individual immunosuppressive drugs (serum trough levels). Serum levels of tacrolimus, everolimus or sirolimus were measured by a rapid sensitive liquid chromatography-tandem mass spectrometry (LC-MS/MS) (26). The therapeutic standard serum-level range is 4–15 μg/l for tacrolimus, 3–8 μg/l for everolimus, and 4–12 μg/l for sirolimus, respectively.

### Statistics

Data are given as boxplots in Tukey style or as means with standard deviation. The proof of normal distribution of our data was performed with the Shapiro-Wilk normality test. For comparison of the serum trough levels of the immunosuppressive drugs we used a two-tailed paired *t*-test. Statistical significant differences of the serum levels of bilirubin were analyzed with a Wilcoxon test. The graphs and analyzes were performed with GraphPad Prism 7. Statistical significance was defined as *p* < 0.05 (confidence level 95%).

### Ethics

This project was approved by the ethic committee of the University Medical Center of Goettingen. The approval number of the institutional review board is 21/7/17An.

## Results

After analysis of the patient records of all 178 liver transplant recipients we could identify 40 patients after LTX with clear time points where a continuous oral therapy with 40 mg pantoprazole once daily was initiated or stopped (Table 1). These patients received immunosuppressive drugs for 1,214 days in the mean (median 787 days) and in a constant dosage during the observation period around the change of pantoprazole medication. The mean age of these patients was 55.1 years (19–75 years, median 57 years). The mean period after liver transplantation was 1,378 days (median 1,169 days). The major indication for liver transplantation in these 40 patients was post-alcoholic liver cirrhosis (*n* = 10 patients) followed by different viral hepatitis (*n* = 9 patients) and hepatocellular carcinoma (*n* = 8 patients). The other patients were transplanted because of metabolic disorders, polycystic liver disease or other hepatic disorders. No patient suffered from rejection or severe infection e.g., pneumonia, biliary infection, gastrointestinal infection, urinary infection, opportunistic infection, or viral infections like cytomegalic virus or other herpes viruses.

**Table 1:** Patient collective.

	**tac**	**eve**	**sir**
Observed patients	30	7	3
Pantoprazole started	10	3	0
Pantoprazole stopped	20	4	3
Mean dosage of immunosuppressive agent (mg/d) Dosage range (mg/d)	5.13 (1–17)	3.43 (2–6.5)	0.83 (0.5–1)

*Characterization of 40 liver transplant recipients with different immunosuppressive drugs and oral co-administration of pantoprazole (40 mg once daily)*.

*eve, everolimus; sir, sirolimus; tac, tacrolimus*.

Thirty patients had an immunosuppressive therapy with tacrolimus. By proving the patient records we identified 10 time points with start of a continuous oral co-administration of 40 mg of pantoprazole once daily (all of them had immediate-release tacrolimus formulation) and 20 time points where a continuous co-administration was stopped (8 patients got extended-release tacrolimus formulation, 12 patients got immediate-release tacrolimus formulation).

In the everolimus group (*n* = 7) we discovered three patients with time points where a constant pantoprazole co-administration was started and four where it was stopped, respectively.

In the sirolimus group (*n* = 3) only three time points with stop of pantoprazole co-administration could be documented.

14/40 patients (35%) got an additional immunosuppressive medication with mycophenolic acid (MMF). The serum levels of MMF were not measured. The most used co-medications in the observed patients was ursodesoxycholic acid (20/40 patients, 50%), followed by the calcium-antagonist amlodipine and the beta-receptor antagonist metoprolol (each 9/40 patients, 22.5%; Supplemental Table 1). Amlodipine can potentially increase the serum levels of tacrolimus, while simvastatin can increase the serum levels both of tacrolimus and sirolimus (27–29). Therefore, when amlodipine (9/40 patients) or simvastatin (3/40 patients) was co-administered only patients treated with a constant dosage of these drugs were included for this evaluation.

**Figure 1:**
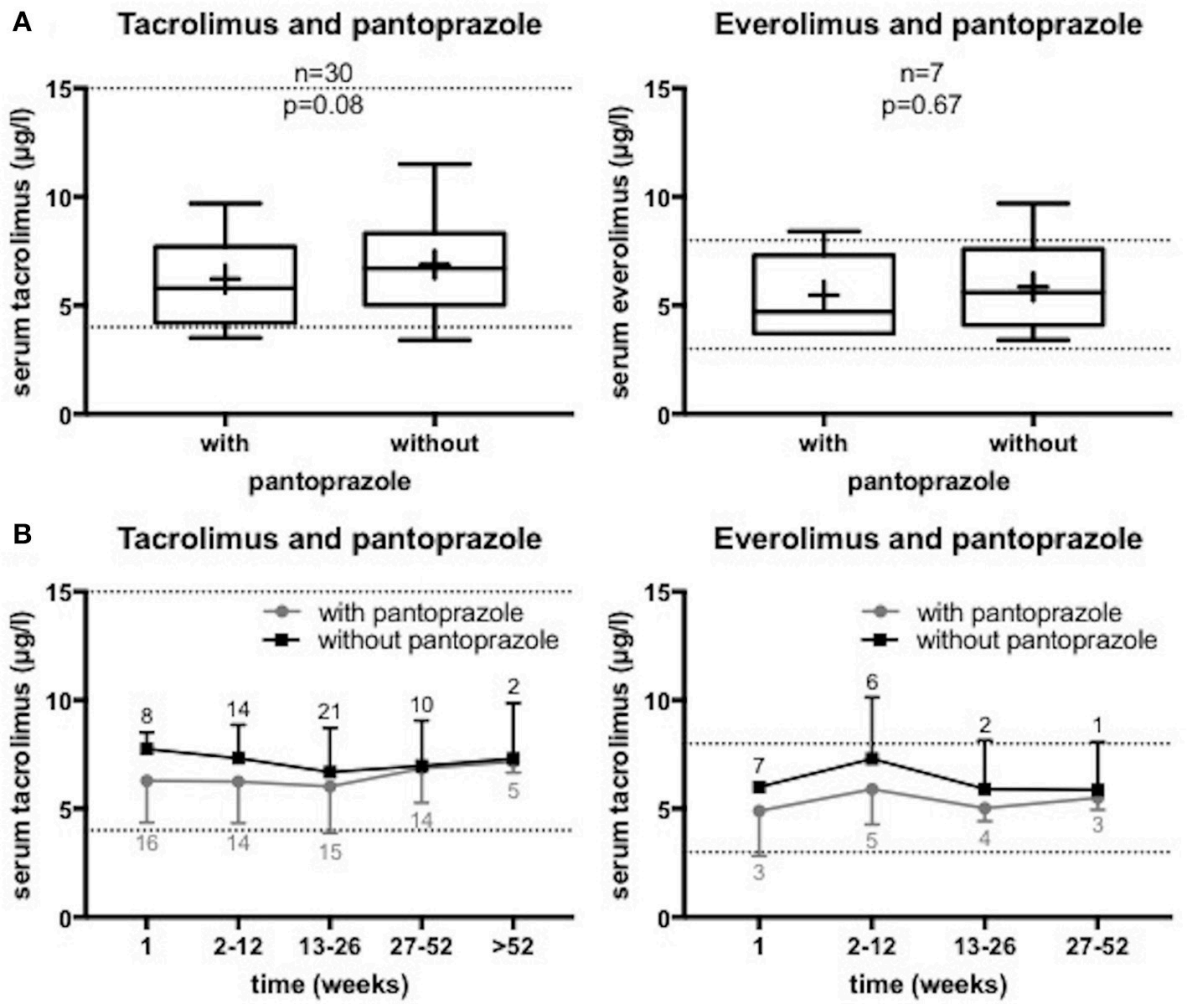
Serum levels of the immunosuppressive drugs tacrolimus and everolimus in the presence or absence of pantoprazole in liver transplant recipients. We analyzed the serum trough levels of the immunosuppressive drugs around start or stop of a continuous oral pantoprazole therapy with 40 mg once daily. All included patients got constant co-medication and constant immunosuppressive drug dosage. The dashed lines mark the therapeutic range of the serum trough levels. **(A)** Analysis of the serum levels measured before and after change of a pantoprazole therapy. The mean observation period was 6.5 ± 8.8 weeks in the tacrolimus group (*n* = 30) and 7.8 ± 7.9 weeks in the everolimus group (*n* = 7), respectively. Data are shown as boxplots in Tukey style. + = mean value. **(B)** Analysis of the serum levels depending on admission or omission of pantoprazole over a period of at least 52 weeks before and after change of pantoprazole co-administration. All routine measurements of the serum trough levels with and without PPI co-administration during the observation period are demonstrated. Data are shown as means with standard deviation of the number of patients indicated.

The serum trough levels of tacrolimus as well as of everolimus in the presence or absence of oral pantoprazole (40 mg once daily) are shown in Figure 1. All observed patients needed no change of dosage of their immunosuppressive agent. There was no significant change of the immunosuppressive drug levels depending on the presence or absence of pantoprazole (tacrolimus group: median drug level 5.8 vs. 6.7 μg/l, *n* = 30 patients, *p* = 0.08; everolimus group: median drug level 4.7 vs. 5.6 μg/l, *n* = 7, *p* = 0.67). The sirolimus group was too small (*n* = 3 patients) for a valid statistical analysis. The median drug level of sirolimus accounted to 4.7 μg/l in the presence of pantoprazole and 5 μg/l in the absence of this PPI, respectively.

The subanalysis of the serum drug levels of tacrolimus applied as extended-release formulation (*n* = 8 patients, *p* = 0.58) or immediate-release formulation (*n* = 22 patients, *p* = 0.08) revealed no significant differences.

It is known that tacrolimus, everolimus and sirolimus are biliary excreted (24, 25). The analyzed patients showed normal total bilirubin levels at the observed time points of measurements of immunosuppressive drug levels.

## Discussion

Pantoprazole did not significantly affect the serum trough levels of the immunosuppressive drugs tacrolimus and everolimus in liver transplant recipients, respectively.

The serum trough levels of tacrolimus as well as of everolimus remained constant during an observation period of more than 52 weeks independent of the presence of pantoprazole (40 mg once daily). These are the first data on the influence of pantoprazole on serum trough levels of everolimus in liver transplanted patients.

Lorf et al. (23) reported a failing effect of pantoprazole on tacrolimus serum levels in two liver transplanted patients. The present data confirm these results in a significantly larger cohort of liver transplant recipients.

As mentioned above, pantoprazole is mainly metabolized by CYP2C19 but also by CYP3A4 (19). Nevertheless, a subgroup of patients with decreased CYP2C19 activity (e.g., heritable poor metabolizers, patients with liver cirrhosis) or patients with absent CYP2C19 enzyme activity which is described in 3% of Caucasians and about 12–22% of Orientals might suffer from drug interactions (30). In these ethnic groups CYP3A4 depending metabolism of pantoprazole might be significantly increased like it is shown for omeprazole (8, 31). Thus, the serum levels of tacrolimus, everolimus or sirolimus could probably be affected by pantoprazole in these cases.

The inhibition of gastric acid secretion by PPIs and the consecutive increased gastric pH can influence the resorption of several drugs (7, 32–34). However, the demonstrated constant serum trough levels indicate no influence in the resorption of various immunosuppressive drugs in our cohort.

Despite of the interaction safety of pantoprazole with the immunosuppressive drugs tacrolimus, everolimus or sirolimus in liver transplant recipients the long time treatment effects of PPIs should be critically observed regarding possible side effects (35). Due to unwanted long-term side effects PPI treatment should be subject to strong indications.

Our analysis is based on retrospective data analysis on patients which were treated in an outpatient basis. To further confirm our findings prospective studies would be appreciated. Additional consideration of the CYP activity and the metabolism including the AUC would be of interest.

Our analysis provides first evidence that the co-administration of pantoprazole does not influence the serum trough levels of tacrolimus, everolimus or sirolimus in liver transplant recipients. Regardless, serum trough levels should be monitored regularly especially when potentially interacting co-medication is altered. These data indicate that the use of pantoprazole in liver transplant recipients seems not to influence immunosuppressive drugs in a clinically relevant way.

## Author contributions

SB, LR, and HS designed the concept. SB, LR, MS, and MH participated in the performance of the project. SB and MS performed the data analysis. SB prepared the manuscript. LR, TL, VE, and HS revised the manuscript. VE approved the final version. SB and LR contributed equally as first authors.

### Conflict of interest statement

The authors declare that the research was conducted in the absence of any commercial or financial relationships that could be construed as a potential conflict of interest.
