# Supplementary material for: Pantoprazole Does not Affect Serum Trough Levels of Tacrolimus and Everolimus in Liver Transplant Recipients
Source: Front Med (Lausanne). 2018 Nov 19;5:320. doi: 10.3389/fmed.2018.00320 (PMC6253821; doi:10.3389/fmed.2018.00320)
Supplement: Supplementary file 1 [file Table_1.docx]

**Supplemental Table 1: Co-medication.** Co-medications of the observed patients in descending order. Only drugs that were used in more than one patient are shown. All drugs were proved for potential drug interactions. Only patients with constant use of potentially interacting drugs were included in this analysis.

|  | **tac**  **n = 30** | **eve**  **n = 7** | **sir**  **n = 3** |
| --- | --- | --- | --- |
| ursodesoxycholic acid | 16 | 3 | 1 |
| mycophenolic acid (MMF) | 12 | 2 | 0 |
| amlodipine | 6 | 2 | 1 |
| metoprolol | 7 | 1 | 1 |
| acetylsalicylic acid | 4 | 2 | 1 |
| prednisolone | 5 | 0 | 0 |
| calcium | 2 | 1 | 1 |
| lamivudine | 2 | 1 | 1 |
| magnesium | 2 | 2 | 0 |
| levothyroxine | 3 | 0 | 0 |
| simvastatin | 1 | 2 | 0 |
| furosemide | 2 | 0 | 0 |
| torsemide | 0 | 2 | 0 |
| pancrelipase (Creon) | 1 | 1 | 0 |
| allopurinol | 1 | 1 | 0 |
| hydrochlorothiazide | 1 | 1 | 0 |
| tamsulosin | 2 | 0 | 0 |
| propranolol | 1 | 1 | 0 |
| insulin | 0 | 2 | 0 |
| gabapentin | 1 | 1 | 0 |
| ramipril | 2 | 0 | 0 |

eve: everolimus, sir: sirolimus, tac: tacrolimus
